# Supplementary material for: Size Reduction to Enhance Crystal-to-Liquid Phase Transition Induced by E-to-Z Photoisomerization Based on Molecular Crystals of Phenylbutadiene Ester
Source: Materials (Basel). 2024 Jul 24;17(15):3664. doi: 10.3390/ma17153664 (PMC11312889; doi:10.3390/ma17153664)
Supplement: Supplementary file 1 [file materials-17-03664-s001.zip › materials-3091540-supplementary-v4/checkCIF-PLATON page 2.htm]

checkCIF/PLATON page 2


# checkCIF (basic structural check) running

---

  
*Checking for embedded fcf data in CIF ...*   
*Found embedded fcf data in CIF. Extracting fcf data from uploaded CIF, please wait* **.** **.** **.** 

# checkCIF/PLATON (basic structural check)

---

Structure factors have been supplied for datablock(s) d8v22220

THIS REPORT IS FOR GUIDANCE ONLY. IF USED AS PART OF A REVIEW PROCEDURE FOR PUBLICATION, IT SHOULD NOT REPLACE THE EXPERTISE OF AN EXPERIENCED CRYSTALLOGRAPHIC REFEREE.

```
No syntax errors found. CIF dictionary  
Please wait while processing ....  Interpreting this report
```

Structure factor report  
  

**Datablock: d8v22220**


---

|  |  |  |
| --- | --- | --- |
| Bond precision: | C-C = 0.0023 A | Wavelength=0.71073 |

|  |  |  |  |
| --- | --- | --- | --- |
| Cell: | a=15.2382(5) | b=11.8007(4) | c=14.4932(5) |
|  | alpha=90 | beta=90 | gamma=90 |
| Temperature: | 213 K |  |  |

|  |  |  |
| --- | --- | --- |
|  | Calculated | Reported |
| Volume | 2606.19(15) | 2606.19(15) |
| Space group | P c c n | P c c n |
| Hall group | -P 2ab 2ac | -P 2ab 2ac |
| Moiety formula | C14 H14 O4 | ? |
| Sum formula | C14 H14 O4 | C14 H14 O4 |
| Mr | 246.25 | 246.25 |
| Dx,g cm-3 | 1.255 | 1.255 |
| Z | 8 | 8 |
| Mu (mm-1) | 0.092 | 0.092 |
| F000 | 1040.0 | 1040.0 |
| F000' | 1040.59 |  |
| h,k,lmax | 18,14,17 | 18,14,17 |
| Nref | 2556 | 2539 |
| Tmin,Tmax | 0.984,0.988 | 0.680,0.746 |
| Tmin' | 0.984 |  |

|  |  |
| --- | --- |
| Correction method= # Reported T Limits: Tmin=0.680 Tmax=0.746 AbsCorr = MULTI-SCAN |  |

|  |  |
| --- | --- |
| Data completeness= 0.993 | Theta(max)= 25.998 |

|  |  |
| --- | --- |
| R(reflections)= 0.0442( 2068) | wR2(reflections)= 0.1225( 2539) |
| |  |  | | --- | --- | | S = 1.024 | Npar= 166 | |

---

```
The following ALERTS were generated. Each ALERT has the format
       test-name_ALERT_alert-type_alert-level.
Click on the hyperlinks for more details of the test.


---

Alert level C
PLAT242_ALERT_2_C Low    'MainMol' Ueq as Compared to Neighbors of        C13 Check 
PLAT911_ALERT_3_C Missing FCF Refl Between Thmin & STh/L=    0.600         16 Report
PLAT913_ALERT_3_C Missing # of Very Strong Reflections in FCF ....          7 Note  


---

Alert level G
PLAT480_ALERT_4_G Long H...A H-Bond Reported H12B     ..O2       .       2.65 Ang.  
PLAT883_ALERT_1_G No Info/Value for _atom_sites_solution_primary .     Please Do !  
PLAT910_ALERT_3_G Missing # of FCF Reflection(s) Below Theta(Min).          2 Note  
PLAT941_ALERT_3_G Average HKL Measurement Multiplicity ...........        4.8 Low   
PLAT965_ALERT_2_G The SHELXL WEIGHT Optimisation has not Converged     Please Check 
PLAT967_ALERT_5_G Note: Two-Theta Cutoff Value in Embedded .res ..       52.0 Degree
PLAT978_ALERT_2_G Number C-C Bonds with Positive Residual Density.          6 Info  


---

   0 ALERT level A = Most likely a serious problem - resolve or explain
   0 ALERT level B = A potentially serious problem, consider carefully
   3 ALERT level C = Check. Ensure it is not caused by an omission or oversight
   7 ALERT level G = General information/check it is not something unexpected

   1 ALERT type 1 CIF construction/syntax error, inconsistent or missing data
   3 ALERT type 2 Indicator that the structure model may be wrong or deficient
   4 ALERT type 3 Indicator that the structure quality may be low
   1 ALERT type 4 Improvement, methodology, query or suggestion
   1 ALERT type 5 Informative message, check
```

---

---

It is advisable to attempt to resolve as many as possible of the alerts in all categories. Often the minor alerts point to easily fixed oversights, errors and omissions in your CIF or refinement strategy, so attention to these fine details can be worthwhile. In order to resolve some of the more serious problems it may be necessary to carry out additional measurements or structure refinements. However, the purpose of your study may justify the reported deviations and the more serious of these should normally be commented upon in the discussion or experimental section of a paper or in the "special\_details" fields of the CIF. checkCIF was carefully designed to identify outliers and unusual parameters, but every test has its limitations and alerts that are not important in a particular case may appear. Conversely, the absence of alerts does not guarantee there are no aspects of the results needing attention. It is up to the individual to critically assess their own results and, if necessary, seek expert advice. **Publication of your CIF in IUCr journals** A basic structural check has been run on your CIF. These basic checks will be run on all CIFs submitted for publication in IUCr journals (*Acta Crystallographica*, *Journal of Applied Crystallography*, *Journal of Synchrotron Radiation*); however, if you intend to submit to *Acta Crystallographica Section C* or *E* or *IUCrData*, you should make sure that full publication checks are run on the final version of your CIF prior to submission. **Publication of your CIF in other journals** Please refer to the *Notes for Authors* of the relevant journal for any special instructions relating to CIF submission. |

---

**PLATON version of 18/05/2022; check.def file version of 17/05/2022**

|  |
| --- |
| **Datablock d8v22220** - ellipsoid plot |
|  |

---

 Download CIF editor (publCIF) from the IUCr   
 Download CIF editor (enCIFer) from the CCDC   
 Test a new CIF entry 
