# Supplementary material for: Size Reduction to Enhance Crystal-to-Liquid Phase Transition Induced by E-to-Z Photoisomerization Based on Molecular Crystals of Phenylbutadiene Ester
Source: Materials (Basel). 2024 Jul 24;17(15):3664. doi: 10.3390/ma17153664 (PMC11312889; doi:10.3390/ma17153664)
Supplement: Supplementary file 1 [file materials-17-03664-s001.zip › materials-3091540-supplementary-v4/Materials-SM-revison-no highlight.pdf]

# Size Reduction to Enhance Crystal-to-Liquid Phase Transition Induced by *E*-to-*Z* Photoisomerization Based on Molecular Crystals of Phenylbutadiene Ester

# Supplementary Materials

**Yu-Hao Li †, Min Cui †, Yi Gong, Tian-Yi Xu and Fei Tong ***

Key Laboratory for Advanced Materials and Joint International Research Laboratory of Precision Chemistry and Molecular Engineering, Feringa Nobel Prize Scientist Joint Research Center, Frontiers Science Center for Materiobiology and Dynamic Chemistry, School of Chemistry and Molecular Engineering East China University of Science and Technology, 130 Meilong Road, Shanghai 200237, China; yuhao_li@mail.ecust.edu.cn (Y.-H.L.); cuimin2021@163.com (M.C.); 22011017@mail.ecust.edu.cn (Y.G.); xutianyi1108@163.com (T.-Y.X.)

* Correspondence: feitong@ecust.edu.cn

† These authors contributed equally to this work.

# Catalogue


Figure S3 .......... 4

Figure S4 .......... 4

Figure S5 .......... 5

Figure S6 .......... 5

Figure S7 .......... 6

Figure S8 .......... 6

Figure S9 .......... 7

Figure S10 .......... 7

Figure S11 .......... 8

Figure S12 .......... 8

Figure S13 .......... 9

Figure S14 .......... 10

Table S1 .......... 11

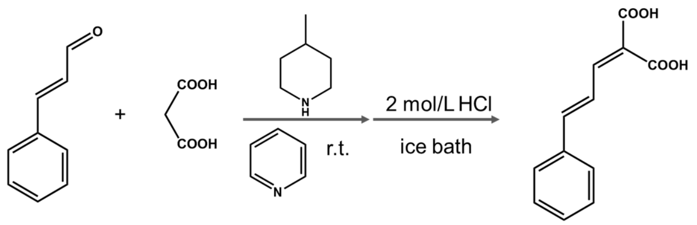


**Figure S1.** The preparation of compound (*E*) -PAPA.

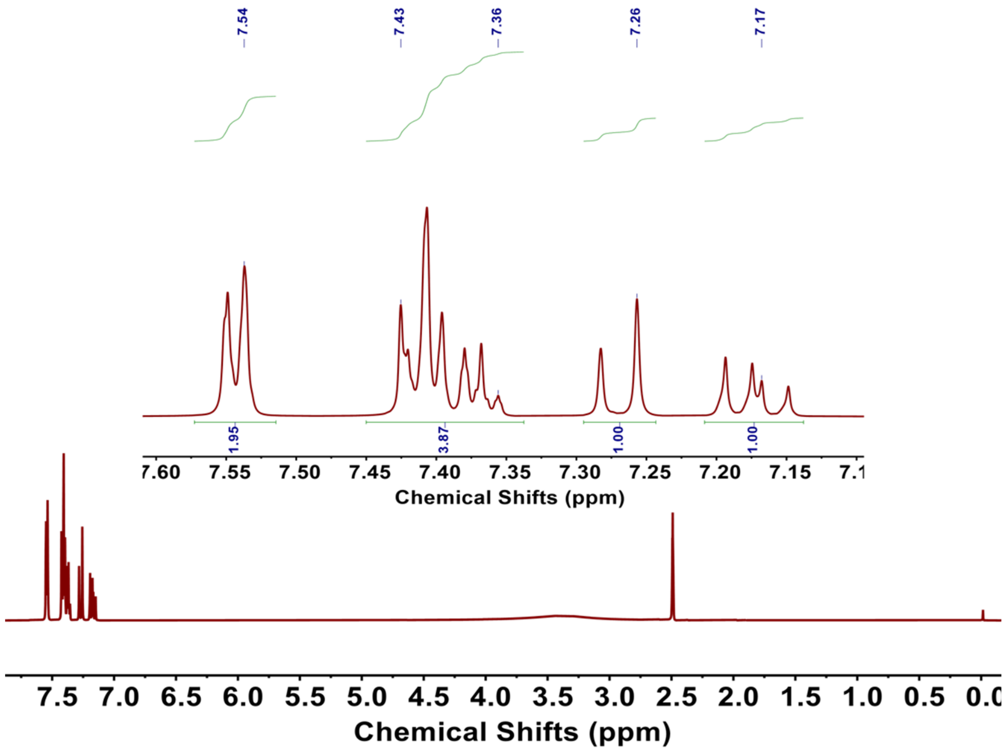


**Figure S2.** $^{1}H$ NMR spectrum of (*E*)-PAPA in DMSO-d6. The peaks at around 2.50 and 3.33 ppm are due to DMSO-d6 and water, respectively. Inset: the magnified region between 7.1 and 7.6 ppm.

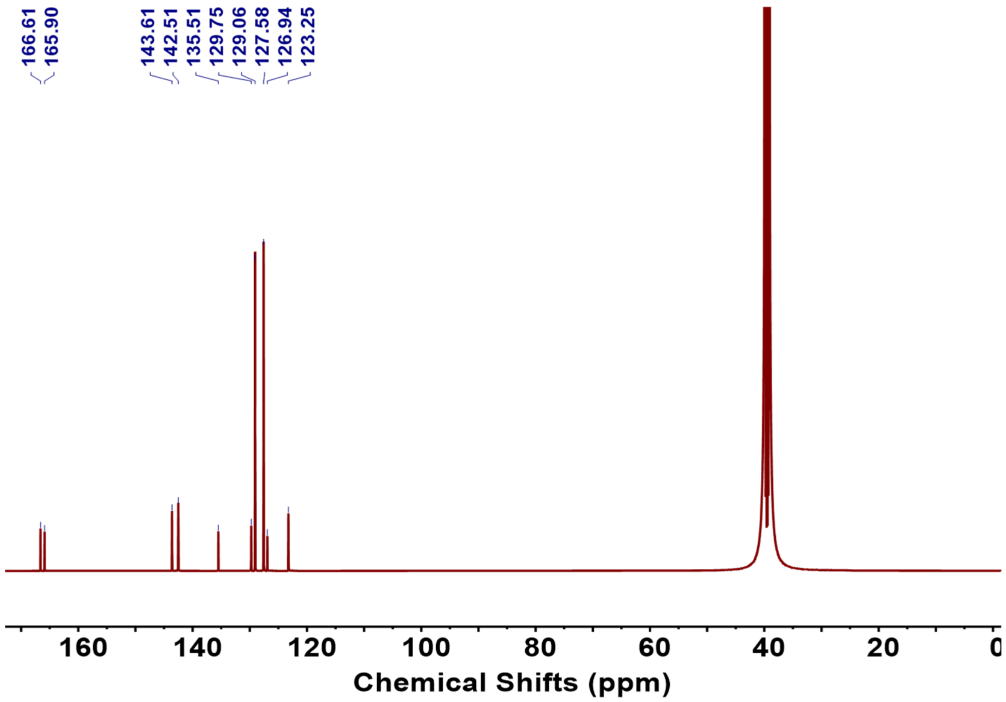


**Figure S3.** $^{13}$C NMR spectrum of (*E*)-PAPA in DMSO-$d_6$. The multiple intense peaks at around 40 ppm are due to DMSO-$d_6$.

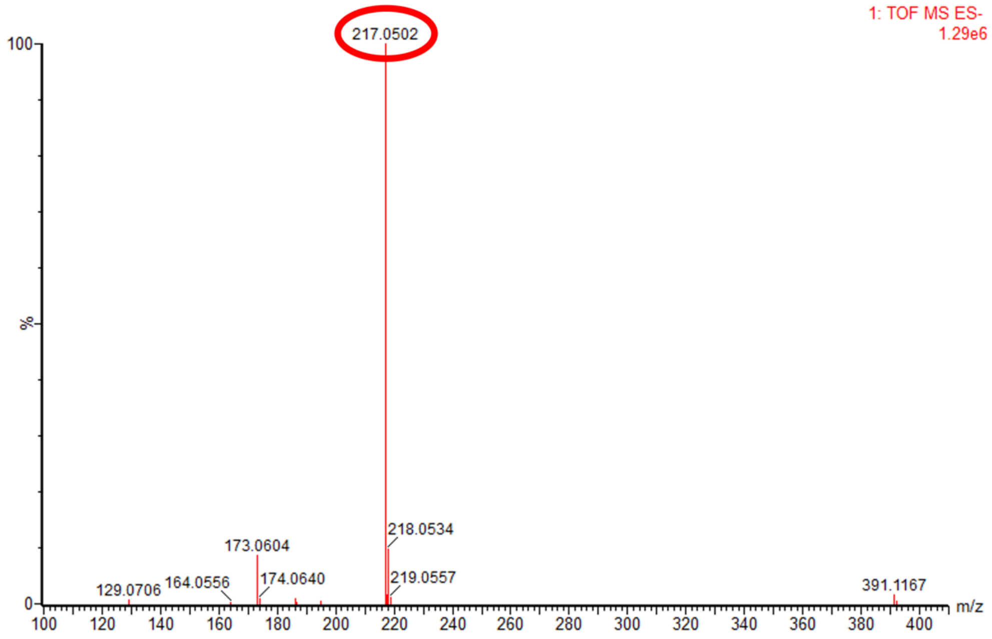


**Figure S4**. HR-MS (ESI) spectrum of (*E*)-PAPA.

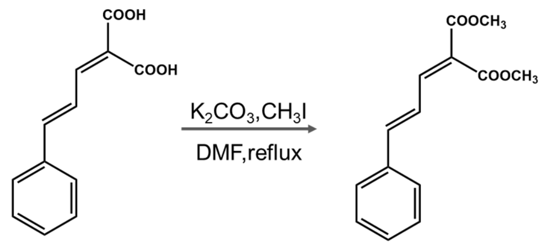


**Figure S5.** The preparation of compound (*E*)-PADM.

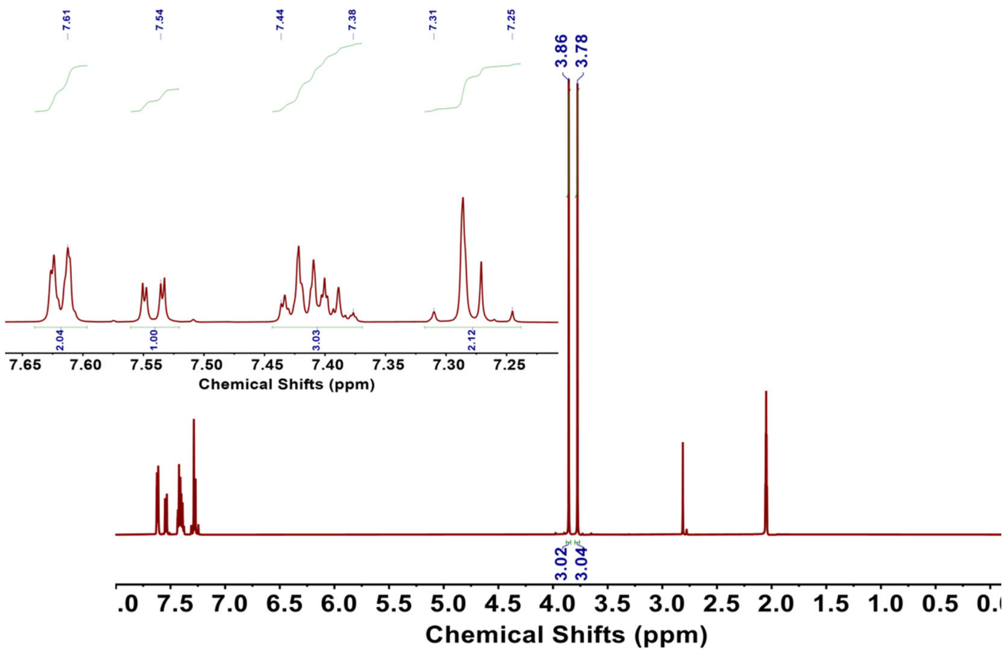


**Figure S6.** $^1H$ NMR spectrum of (*E*)-PADM in acetone-$d_6$. The peaks at around 2.05 and 2.84 ppm are due to acetone-$d_6$ and water, respectively. Inset: the magnified region between 7.2 and 7.7 ppm.

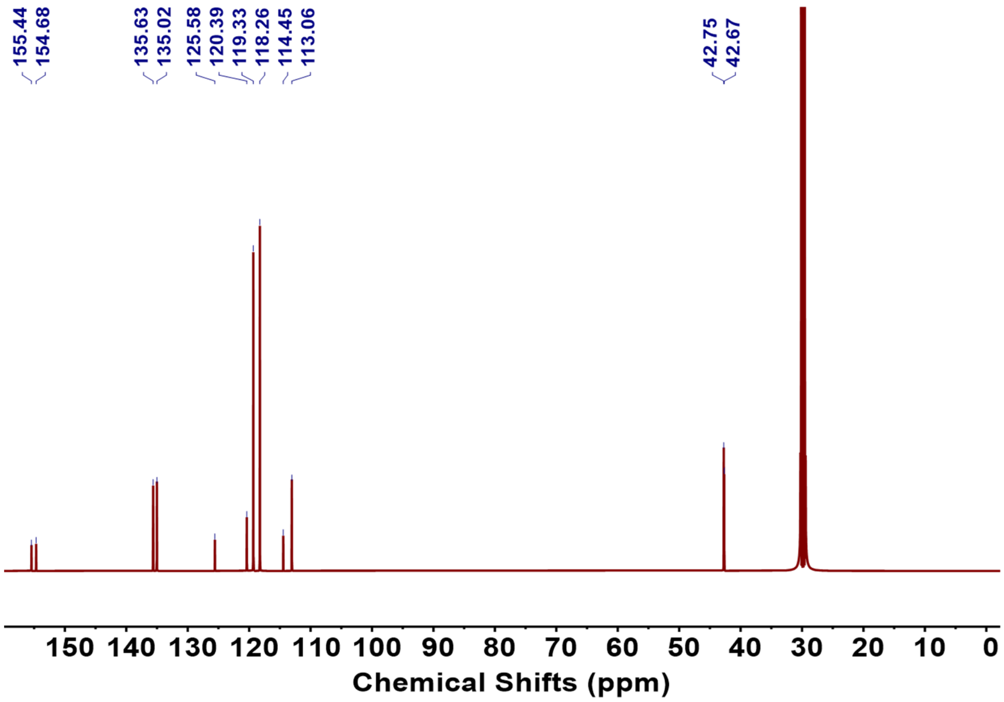


**Figure S7.** $^{13}C$ NMR spectrum of (*E*)-PADM in acetone-$d_6$. The multiple intense peaks at around 30 ppm are due to acetone-$d_6$.

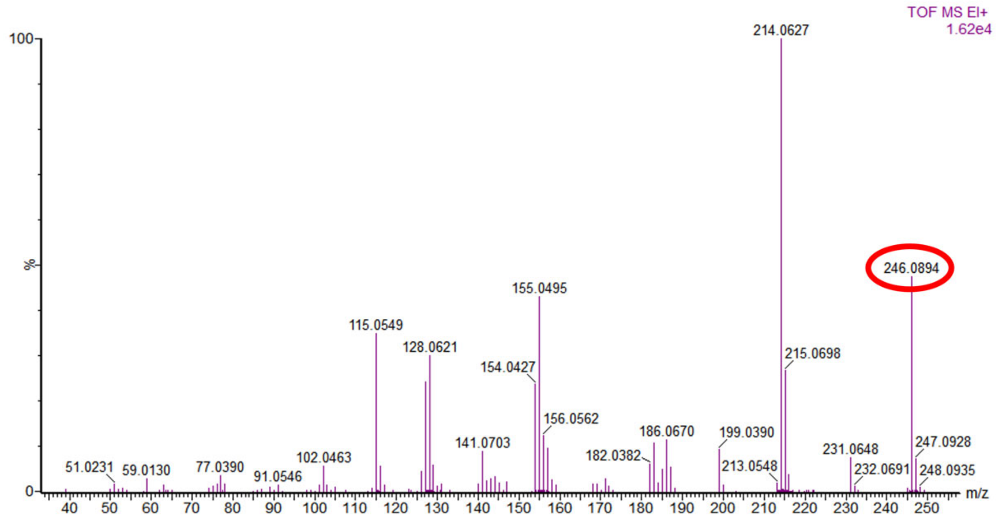


**Figure S8.** HR-MS (EI) spectrum of (*E*)-PADM.

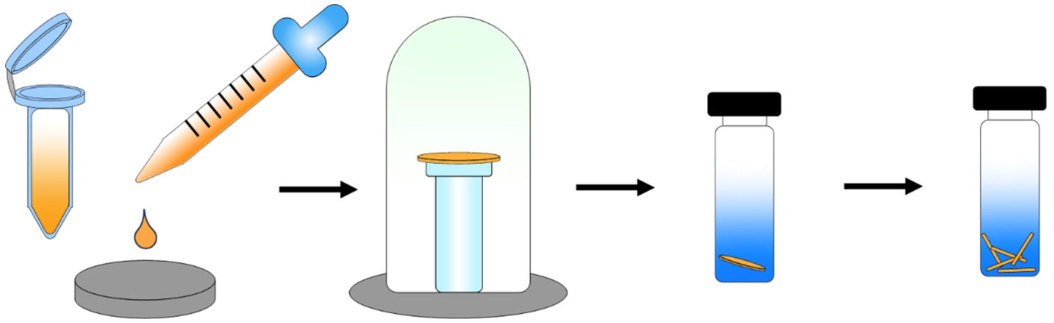

**Figure S9.** Schematic diagram of the process of preparing nanowire crystals by the solvent annealing method in AAO template.

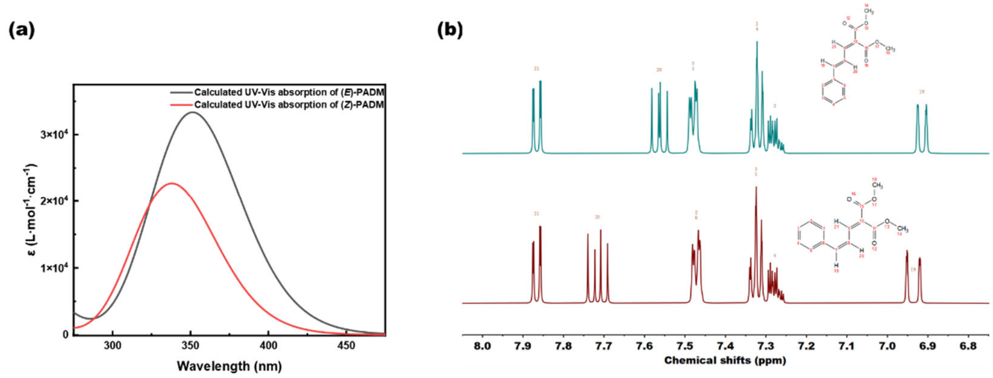


**Figure S10.** (a) Calculated UV-Vis absorption curves of (*E*)-PADM and (*Z*)-PADM using density functional theory (DFT) computational method at 6-31G'basic set. (b) Predicted $^{1}$H NMR of (*E*)-PADM and (*Z*)-PADM by Mestrenova 12.0

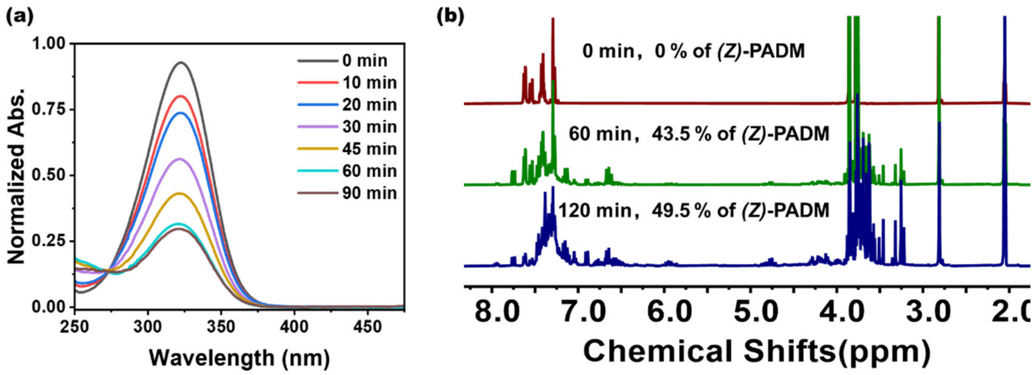


**Figure S11.** (a) UV-Vis absorption spectra of (*E*)-PADM crystal powder dissolved in tetrahydrofuran ($1\times10^{-5}$ M) under the same conditions but with a light intensity of 10.0 mW/cm$^2$; (b) $^1$H NMR spectra of the same (*E*)-PADM sample in acetone-$d_6$ solution (0.041 M) after different irradiation times.

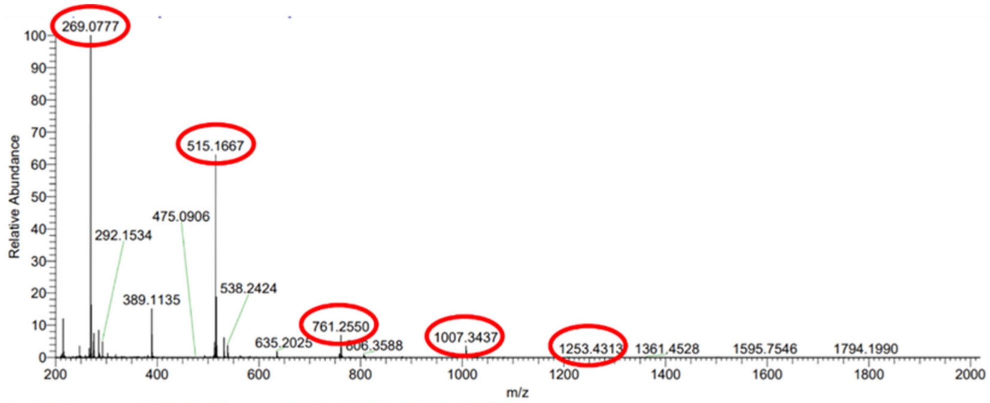


T: FTMS + p ESI Full ms [200.0000-3000.0000]
m/z= 245.59-285.84

| m/z | Intensity | Relative | Theo. Mass | Delta (ppm) | Composition |
|---|---|---|---|---|---|
| 269.0777 | 334874240.0 | 100.00 | 269.0784 | -0.73 | $C_{14}H_{14}O_4Na$ |

**Figure S12**. HR-MS (ESI) spectrum of (*E*) -PADM powder crystal irradiated by 365 nm light (light intensity 500.0 mW/cm$^2$) for 2 h. The red circle marks the peaks of monomers, dimers, etc.

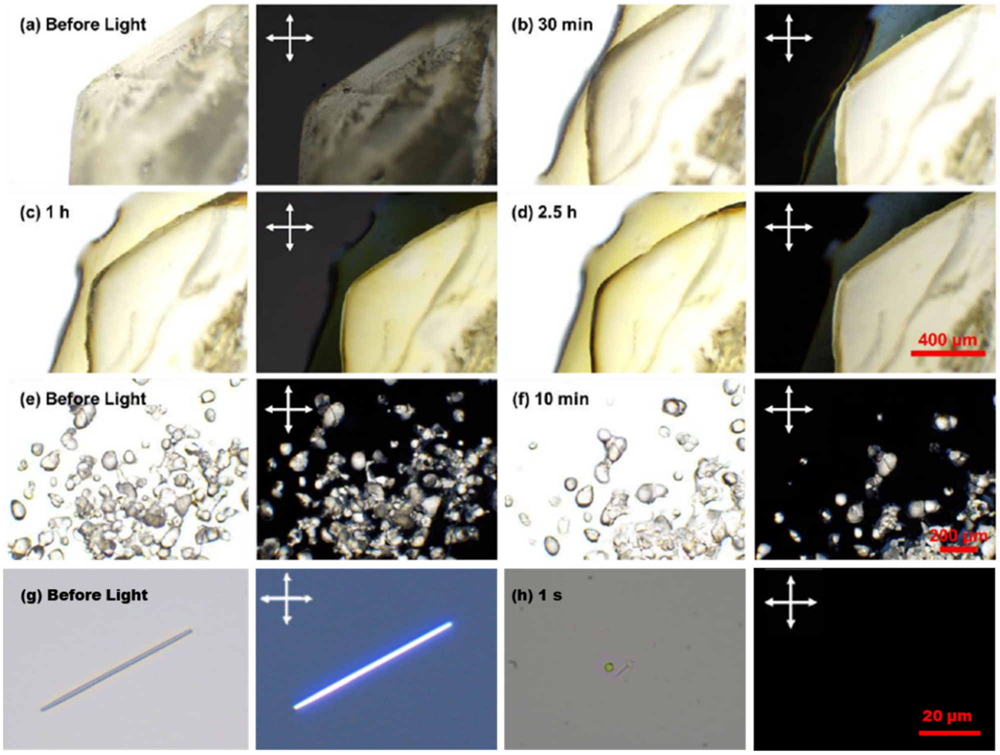


**Figure S13.** Optical microscope and cross-polarized light microscopy images of (*E*)-PADM crystals: (a) Massive crystal before illumination; (b) after 30 min of illumination; (c) After illumination1; (d) After 2.5 hours of illumination; The scale bar is 400 µm. (e) Before light exposure of powder crystals; (f) After 10 minutes of illumination, the scale bar is 400 µm. (g) Before light exposure of nanowires; (f) After 1 second of illumination, the scale bar is 25 µm.

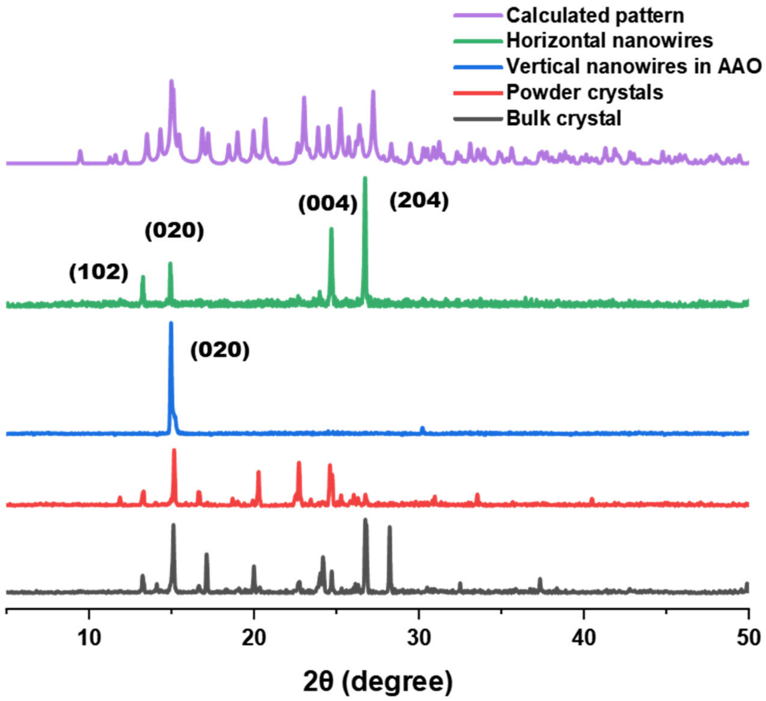


**Figure S14.** (a) The (*E*) -PADM monomer theoretical value calculated from single crystal data (purple line), the PXRD pattern obtained from the (*E*)-PADM nanowire crystals test in the horizontal state (green line), the PXRD pattern obtained from the (*E*)-PADM nanowire crystals test in the vertical state (blue line), the PXRD pattern obtained from the (*E* -PADM powder crystals (red line), the PXRD pattern obtained from the (*E*)–PADM bulk crystals test in the vertical state (black line)

**Table S1.** Crystal data and structure refinement for *(E)*-PADM. CCDC No. 2364020 deposition number: 2364020.

| | | |
|---|---|---|
| Identification code | d8v22220 | |
| Empirical formula | C14 H14 O4 | |
| Formula weight | 246.25 | |
| Temperature | 213(2) K | |
| Wavelength | 0.71073 Å | |
| Crystal system | Orthorhombic | |
| Space group | *P c c n* | |
| Unit cell dimensions | a = 15.2382(5) Å | ◎= 90°. |
| | b = 11.8007(4) Å | ◎= 90°. |
| | c = 14.4932(5) Å | ◎ = 90°. |
| Volume | 2606.19(15) Å$^3$ | |
| Z | 8 | |
| Density (calculated) | 1.255 Mg/m$^3$ | |
| Absorption coefficient | 0.092 mm$^{-1}$ | |
| F(000) | 1040 | |
| Crystal size | 0.180 x 0.150 x 0.130 mm$^3$ | |
| Theta range for data collection | 2.673 to 25.998°. | |
| Index ranges | -18<=h<=18, -11<=k<=14, -17<=l<=17 | |
| Reflections collected | 12165 | |
| Independent reflections | 2539 [R(int) = 0.0281] | |
| Completeness to theta = 25.242° | 99.2 % | |
| Absorption correction | Semi-empirical from equivalents | |
| Max. and min. transmission | 0.7456 and 0.6796 | |
| Refinement method | Full-matrix least-squares on F$^2$ | |
| Data / restraints / parameters | 2539 / 0 / 166 | |
| Goodness-of-fit on F$^2$ | 1.024 | |
| Final R indices [I>2sigma(I)] | R1 = 0.0442, wR2 = 0.1110 | |
| R indices (all data) | R1 = 0.0564, wR2 = 0.1225 | |
| Extinction coefficient | 0.028(5) | |
| Largest diff. peak and hole | 0.377 and -0.303 e.Å$^{-3}$ | |
